# Disease awareness campaigns in printed and online media in Latvia: cross-sectional study on consistency with WHO ethical criteria for medicinal drug promotion and European standards

**DOI:** 10.1186/s12889-018-6202-2

**Published:** 2018-11-28

**Authors:** Teresa Leonardo Alves, Elita Poplavska, Signe Mezinska, Ieva Salmane-Kulikovska, Liga Andersone, Aukje K. Mantel-Teeuwisse, Barbara Mintzes

**Affiliations:** 1 WHO Collaborating Centre for Pharmaceutical Policy and Regulation, Division Pharmacoepidemiology & Clinical Pharmacology, Utrecht Institute for Pharmaceutical Sciences (UIPS), Utrecht University, Utrecht, The Netherlands; 2 Faculty of Pharmacy, Riga Stradins University, Riga, Latvia; 3 Faculty of Medicine, University of Latvia, Riga, Latvia; 4 Faculty of Pharmacy and Charles Perkins Centre, The University of Sydney, Sydney, Australia; 5 Institute of Public Health, Riga Stradins University, Riga, Latvia

**Keywords:** Communications media, Health campaigns, Pharmaceutical industry, Pharmaceutical policy, Consumer health information

## Abstract

**Background:**

European legislation prohibits direct-to-consumer advertising of prescription medicines, but allows drug manufacturers to provide information to the public on health and diseases. Our aim was to measure the frequency of disease awareness campaigns in Latvian media and assess their compliance with international and European standards.

**Methods:**

Materials on health/disease and treatments were collected between April and September 2015 from 12 newspapers and magazines and six online portals. Disease awareness campaigns were assessed using a previously developed instrument based on the WHO Ethical Criteria for Medicinal Drug promotion and European standards (EU law and pharmaceutical industry self-regulatory guidelines). Collected materials were used to examine the information provided on medical conditions and their diagnosis and treatment. The inter-rater reliability was calculated.

**Results:**

We collected 263 materials from print (*n* = 149) and online media (*n* = 114); 94 were news items and 169 were disease-awareness advertisements. Cancer, cardiovascular problems, allergies and respiratory diseases were common topics. Of the 157 campaigns assessed, non-compliance was identified in 149 cases (inter-rater reliability 90%), mainly due to misleading or incomplete information, lack of balance and the absence of a listed author/sponsor. Six disease awareness campaigns directly mentioned a pharmaceutical product by brand name and other four included the logo or name of a manufacturer, referred to a condition and indirectly mentioned a treatment, all in contravention with European law.

**Conclusions:**

The compliance of disease awareness campaigns in Latvian media with international and European standards is low. This raises concerns about the nature of information being conveyed. Through lack of balance, missing sponsorship information, and misleading or incomplete information, these campaigns could contribute to inaccurate self-diagnosis and generate demand among those who might not need medical treatment.

**Electronic supplementary material:**

The online version of this article (10.1186/s12889-018-6202-2) contains supplementary material, which is available to authorized users.

## Background

In countries where direct to consumer (DTC) advertising of prescription medicines is banned, companies are testing the limits of regulatory systems with disease oriented advertising, public relations campaigns and unbranded advertising to the public [1–3]. The approach behind such activities is that mass media expands the patients’ disease and/or drug awareness and motivates them to visit physicians for previously untreated conditions [4]. Promotional campaigns aimed at physicians are often run concomitantly so that practitioners have a specific product in mind when patients ask about new treatments [5].

Proponents of direct-to-consumer communication highlight the need to empower the patient by facilitating access to information which increases knowledge about medicines, diseases and therapeutics [2]. A greater involvement of patients in their treatment could be regarded as contributing to safer consumer choices and improved patient autonomy [6]. Similarly, greater awareness about diseases could lead to better detection, diagnosis and treatment [7]. On the other hand, users might not always be able to judge the information conveyed [2] and campaigns could encourage healthy people to seek unnecessary tests or medication [8]. Campaigns at the time of launch of a new drug could have especially negative implications given the limited evidence available about a drug’s risk profile. Moreover, if the information provided is portrayed as a community service, the public might remain unaware of its commercial intent [5].

One key concern is that campaigns could contribute to overdiagnosis which occurs when people are labelled with or treated for a disease that would never cause them harm, leading to the overuse of further tests and treatments [9]. Overdiagnosis happens in a range of common conditions and appears to be increasing [10]. The pharmaceutical and medical device industries, which aim to maximize health but also has a conflicting interest in expanding product sales, are one of the recognized drivers of overdiagnosis [1, 9].

Disease awareness or condition-oriented campaigns can be effective tools in familiarizing consumers with a disease and a specific pharmaceutical intervention and raise therefore ethical and public health questions similar to those of direct-to-consumer drug promotion [11]. Bearing this in mind, it is pertinent to explore whether the information conveyed in such campaigns is meeting current legal and ethical standards and to distinguish legitimate health information from promotional activities.

Drug manufacturers are legally prohibited from communicating directly with consumers about their prescription-only products, except in New Zealand and in the United States of America [12]. European Union (EU) legislation prohibits direct-to-consumer advertising of prescription medicines as a public health protection measure [13]. However, campaigns to the public about diseases and health from drug manufacturers are allowed, provided there are no direct or indirect references to specific prescription-only medicines [14]. Each member state is then responsible for transposing and implementing the directive. In Latvia, legal provisions on medicines’ advertising define pharmaceutical promotion as “*any form of notification, activity, and measure if the purpose thereof is to promote the prescription, distribution, or use of medicinal products”* [15] but there is no specific guidance about the provision of health and treatment information. The same applies to the national voluntary code of conduct published by pharmaceutical manufacturers’ associations on which national self-regulatory mechanisms are based [16].

While the literature on disease awareness campaigns is relatively scarce and comes from industrialized countries, there is some evidence that such activities increase awareness of the advertised conditions, as well as rates of consultations and prescriptions of the sponsored product [17–19]. Research in Australia and the Netherlands also suggest that exposure to this type of campaigns is relatively common [20, 21]. In a previous study, we developed an instrument to assess the compliance of printed disease awareness campaigns in the Netherlands with international and Dutch regulations [21]. Although this was a small pilot study over a short study period, it identified an alarming lack of compliance of disease awareness campaigns in Dutch printed media with the WHO Ethical criteria for medicinal drug promotion [22] and with national pharmaceutical industry self-regulatory guidelines [23], as well as some evidence of likely contraventions of EU legislation.

We aim to use that same instrument over a longer period in another EU member state – Latvia - where significantly less resources are devoted to health and out-of-pocket payments for health are among the highest when compared to other countries in the Organisation for Economic Cooperation and Development [24]. This study measures the frequency of health and treatment information in printed and online media in Latvia and compares the information provided in news items and disease awareness campaigns. It also assesses the compliance of disease awareness campaigns with the WHO Ethical Criteria and European standards (namely European Union law and the pharmaceutical industry self-regulatory guidelines for Information on Prescription-only medicines). This is the first study to examine disease awareness campaigns in the Baltic Region.

## Methods

### Selection and coding of materials

Data collection took place from April to September 2015. We selected the three top print and online media targeting a varied audience in Latvia, available in either Latvian or Russian language, based on high circulation and subscription numbers from publicly available reports [25–27]. These included: three daily and three weekly newspapers; three monthly and three health magazines; three news and three health portals (see Additional file 1: Table S1). All were accessed at public libraries and available either in Latvian or Russian. In Latvia, 37.2% of the population are Russian-speaking and media are available in both languages [28].

From all the items covering health topics identified in the various media, we selected materials which mentioned conditions or symptoms or manufacturers and provided treatment suggestions (either directly or indirectly). The full inclusion criteria followed the methodology of a previous study [21] and were based on an interpretation of legal provisions [14], which prohibit direct and/or indirect reference to a pharmaceutical product. From all the items covering health topics identified in the various media, we selected materials which mentioned conditions or symptoms or manufacturers and provided treatment suggestions (either directly or indirectly). Materials on issues governed by different regulations such as nutraceuticals, homeopathic products, over-the-counter medication and vaccines were excluded. Prior to data collection, a training session was conducted on application of inclusion criteria and the instrument, with methods piloted during late 2014 and early 2015. Three researchers (EP, SM, LA) then selected materials published between April and September 2015, with duplicate independent screening of all included media and any disagreements resolved by consensus.

### Classification of materials: Identifying disease awareness campaigns

We separated the collected materials into two groups:

- Group I were news items with listed authors or attributed to a news desk. These were not assessed using the tool as reports by the press to the public are not subject to regulations or guidelines on pharmaceutical promotion.
- Group II were disease awareness campaigns without a listed author. These were scored using the instrument described below.

We extracted general and key content characteristics for both Group I and Group II materials on the following factors:

- *publication type:* subscription status (paid or free); language; frequency;
- *author* (yes/no);
- *content*: non-drug options mentioned; physician referral; reference to clinical expert or spokesperson; referral to patient organization or support group; one or more brand-name drugs recommended; availability of new treatment noted; referral to a website; company’s name or logo listed; sponsored by a clinic or hospital.

### Assessing compliance of disease awareness with guidelines

We applied an instrument developed in a previous study [21] and based on seven relevant criteria from the WHO Ethical Criteria for Medicinal Drug Promotion [22] and Dutch pharmaceutical industry self-regulatory guidelines [23]. These include use of: promotional information; misleading or incomplete information; fear; inadequate language; lack of balance; testimonials; and absence of source/author. Dutch and Latvian self-regulatory codes on pharmaceutical promotion are subject to EU regulations [14] and are generally similar in approach as they are both based on the self-regulatory codes issued by the European Federation of Pharmaceutical Industries and Associations (EFPIA) [29]. Additional file 2: Table S2 describes the overlap between relevant provisions in international guidelines and the instrument’s domains. Websites that were mentioned in disease awareness campaigns were also assessed using the instrument and the results of that assessment are presented separately. Three authors (EP, LA, ISK) independently pilot tested the instrument on a sample of materials (*n* = 20). Materials were duplicate coded and differences in scoring resolved through consensus.

### Statistics

Descriptive statistics are presented and risk ratios (RR) were calculated comparing frequencies of information provision in news items (Group I) and disease awareness campaigns (Group II). Inter-rater reliability was measured using the intraclass correlation coefficient two-way random effects model [30]. We used chi-square to test for differences by language (reported jointly if similar). Frequency of non-compliance per key criteria was compared between the Latvian campaigns and those of a previous Dutch study [21]. Data analysis was conducted using SPSS version 24.

## Results

### Assessing disease-awareness frequency in media

**Fig. 1:**
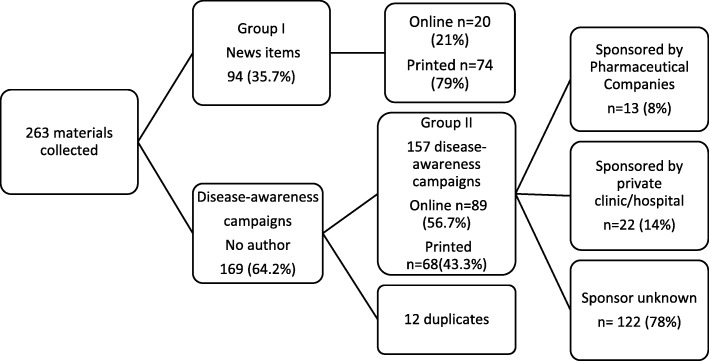
Materials collected and their allocation per type of media and sponsor, when applicable

A total of 263 materials were collected, 94 (35.7%) of which were news items (Group I) and 169 (64.2%) were disease awareness campaigns to be scored by the instrument (Group II) (see Fig. 1). This means that on average, ten materials covering disease and treatment topics were published in print or online media every week, 6 of which were disease awareness campaigns. We identified 12 duplicate disease awareness campaigns within Group II, which were excluded for all other analyses. Three media sources (*n* = 3) contained no materials on health and treatment. Results are presented jointly for Latvian and Russian media as information frequency did not differ significantly by language. The most common topics in news items were dermatological problems (12.8%), cancer (11.7%), cardiovascular diseases (9.6%), pain (9.6%), and gastrointestinal disorders (5.3%). Within disease awareness campaigns, the most frequent themes were cardiovascular diseases (10.7%), dermatological problems (8.3%), cancer (7.7%), urological problems (7.7%), and pain (7.1%).

As is described in Table 1, news items included quotes from key opinion leaders more often, and mentioned the availability of a new treatment, whereas disease awareness campaigns were more likely to refer viewers to a website (RR = 4.04, 95%CI 1.46;11.19) or a pharmaceutical company (RR = 7.78, 95%CI 1,03;58.55). These disease-awareness campaigns were also often sponsored by a hospital or clinic (RR = 3.29, 95% CI 1.17;9.26). Nearly all the materials recommended seeing a physician. Quoted key opinion leaders were most frequently general practitioners and leading specialist physicians from academic hospitals, such as cardiologists and gastroenterologists. Non-drug or lifestyle interventions were mentioned over half the time, although this occurred more often in news items than in disease awareness campaigns. The type of lifestyle interventions mentioned most frequently were exercise (e.g. for depression, varicose veins, pain, urological problems); and psychotherapy (e.g. in cases of depression, compulsive eating, vegetative dystonia).

**Table 1:** Frequency of information provided across materials and their Risk Ratio

Information included	Group I News Items (*n* = 94) (% within group)	Group II Disease Awareness Campaigns (*n* = 157) (% within group)	Risk Ratio (RR) Disease awareness campaigns versus news items (95% CI)
Suggestion to visit a physician	86 (91.5%)	132 (84.1%)	0.92 (0.883;1.01)
Key opinion leader or public figure quoted	89 (94.7%)	94 (59.9%)	0.63 (0.55;0.72)
Non-pharmaceutical interventions in addition to therapy	67 (71.3%)	86 (54.8%)	0.77 (0.63;0.93)
Referral to a website	4 (4.3%)	27 (17.2%)	4.04 (1.46;11.19)
Sponsorship by specific clinic	4 (4.3%)	22 (14.0%)	3.29 (1.17;9.26)
Mention of availability of a new medicine or treatment option	17 (18.1%)	16 (10.2%)	0.56 (0.30;1.06)
Pharmaceutical company name or logo	1 (1.1%)	13 (8.3%)	7.78 (1.03;58.55)
Patient organization or support group	4 (4.3%)	9 (5.7%)	1.35 (0.43;4.25)
Brand-name pharmaceutical product	5 (5.3%)	6 (3.8%)	0.72 (0.22;2.29)

### Compliance of the disease awareness campaigns with guidelines

Of the 157 diseases awareness campaigns assessed, 149 (94.9%) were non-compliant. Inter-rater agreement for independent coding of judgments of compliance with guidelines was high: 0.906 [95% CI 0.877; 0.929]. Non-compliance was most often due to the absence of author or source (*n* = 131, 78%), use of misleading or incomplete information (*n* = 61, 36%), or lack of balance (*n* = 58, 35%). In total, 29.9% (*n* = 50) of campaigns were non-compliant with two criteria and 19.1% (*n* = 32) with three criteria.

Table 2 provides some examples of non-compliance per key criteria and campaign topic.

**Table 2:** Examples of non-compliance per key criteria from the disease awareness campaigns

Key criteria	Problem identified	Example *(condition)*
Promotional information	Reference to pharmaceutical products to treat a condition or disease in combination with: - the name, logo and website of a pharmaceutical company; - or a website for a disease awareness campaign; - or quick response codes to dedicated websites.	“For example, one of the current treatments recommended by doctors for premature ejaculation is a serotonin reuptake inhibitor which prolongs intercourse for men older than 18 for up to 200–400%”. The name and logo of a pharmaceutical company as well as a dedicated website are mentioned. *(Premature ejaculation)* [32]
		“My doctor informed me about a compassionate use program in which 17 patients with hepatitis C had an opportunity to receive the new non-interferon therapy for free, which guaranteed 97–100% cure rate. […] The program was supported by pharmaceutical company X”. *(Hepatitis C)* [21]
		A website about upper respiratory tract conditions states: “inhaled corticosteroids are the most effective bronchial asthma therapy”. The website includes the logo and the name of an asthma medication manufacturer (*Asthma*, website, LV).
		“Selective progesterone receptor modulator is the approved pharmaceutical treatment for uterine fibroids. It reduces bleeding and fibroid volume.” The website includes the logo of a pharmaceutical company, as well as a section for specialists where the product’s brandname is mentioned (*Uterine fibroid*, website, LV)
Misleading or incomplete information	No reference is provided to the sources of information provided about prevalence of disease.	“Approximately 90% of the world population suffers from lower back, neck line and muscle pain…” (*Back pain, RU)*
		“Every 30 s someone has a fracture due to osteoporosis”. *(Osteoporosis, RU)*
		“After reaching 60 years of age, approximately 60% of population suffers from venous insufficiency” *(Varicose veins, LV)*
		“It is possible that you are among the 90% of the population who suffer from herpes blisters. Here is the information that you need to know about the herpes virus.” (*Herpes*, LV)
		“Approximately half of ovarian cancer cases are lethal. This is due to the asymptomatic nature of the cancer and delayed diagnosis” (*Cancer*, LV*)*
Use of fear	Reference to disability caused by the disease, either through text or picture.	“If left untreated hemorrhoids will only get worse – inflamation will develop into abcess, pain will increase, bleeding and prolapse will form thrombs.” *(Hemorrhoids, RU)*
		“If Lyme disease is not diagnosed and treated in a timely manner, other symptoms of Lyme disease can develop several weeks, months or years after the tick bite, such as arthritis, nervous system or cardiovascular disorders” *(Lyme disease, LV)*
Inadequate language	Uses medical terminology	“[High blood pressure] also increases the risk of heart diseases - ischemic heart disease, cardiomyopathy, development of infarction and stroke.” *(Cardiovascular disease, LV)*
Lack of balance	More emphasis on the benefits of pharmaceutical treatment than risks. Symptoms are accentuated by layout and/or enumeration. Risk factors are portrayed as diseases. Treatment is accentuated.	“Botulin injections are one of the most effective methods to fight excessive sweating. […] The effect will appear on the 4th to 6th day after the injection and will last six to 9 months. ”(*Excessive sweating*, LV).
		Symptoms are referred to in headings in big and bold typeface. *(Diabetes, Nr 105, LV) (Asthma, Nr 30) (Alzheimer, Nr 138, LV)*
		“There are several tablets you can use for the treatment of erectile dysfunction. […] There is a high chance that treatment will work (in 8 cases out of 10 treatment is effective). Please discuss the advantages and disadvantages of these treatments with your doctor.” (*Erectile dysfunction* website, LV)
Use of testimonials	Specialist mentions treatment and specific drug classes	Quote from a general practitioner “If you have frequent and pronounced herpes infections you will need to use acyclovir [tablets] – a serious medication in high doses.” (*Herpes simplex infection*, Nr. 9, LV)
	A comparison is made of the patient’s experience before and after treatment with a specific drug.	“I trust my doctor a lot but I was still worried that [with a new therapy] I would experience the same side effects I had before... This time everything was different! I only had to worry about taking my pills on time.” *(Hepatitis C, Nr 52, LV)*
Absence of author and/or sponsor	No author and/or sponsor identified.	Conditions or Symptoms where this non compliance was identified: *Anemia* *Alzheimer* *Diabetes,* *Cancer (breast, cervical, colorectal, melanoma)* *Cough* *Contraception* *Glaucoma* *Gout* *Excessive sweating* *Eye infection* *Eating disorders* *Female sexual dysfunction* *fibromioma* *Heart failure* *Heart attack* *Hemorrhoids* *Hepatitis C* *Herpes simplex* *Hypertension* *Lyme disease* *Migraine* *Nail fungus* *Osteoporosis* *Pain* *Parkinson* *Psoriasis* *Pulmonary arterial hypertension* *Seasonal affective disorder* *Smoking cessation* *Sport related injuries* *Stroke* *Seasonal allergy* *Trophic ulcers* *Varicose veins* *Warts*

**Fig. 2:**
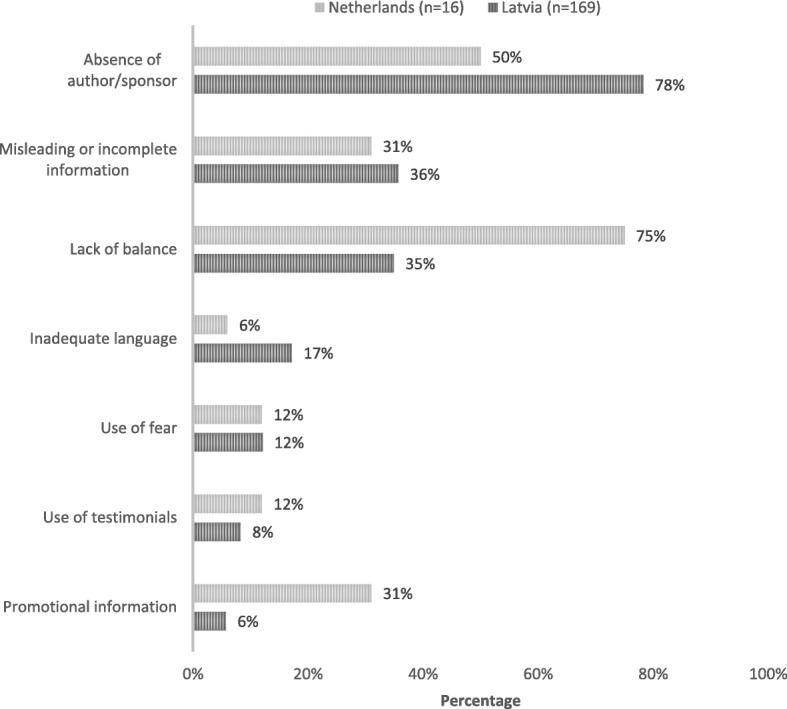
Non-compliance of disease awareness campaigns per key criteria*. *See Additional file 2: Table S2 for operational definitions of the key criteria

Figure 2 provides an overview of compliance levels per key criteria, and compares results with those obtained in the Dutch study [21]. The Latvian campaigns seemed overall more compliant with standards than the Dutch but were more likely to not to mention an author/sponsor, contain misleading or incomplete information or inadequate language.

Twenty-three of the 157 campaigns (14.6%) listed dedicated websites, 20 (86,9%) of which were also non-compliant with guidelines. Eight of these websites were sponsored by a pharmaceutical company. Ten disease-awareness campaigns (6.4%) were likely in contravention of European law: four included the logo or name of a pharmaceutical company and both referred to a condition and mentioned a treatment indirectly; six mentioned a medicine by its brand name.

## Discussion

Our study confirms that there is a strong focus on health and treatment information in Latvian media with more than ten items being published every week covering various topics, including both health-related news items and disease-awareness campaigns. An average of six disease-awareness campaigns were published per week, which is a higher frequency than that reported in similar studies in the Netherlands and in Australia [20, 21].

In this sample, the overwhelming majority of Latvian disease-awareness campaigns (94.9%) did not comply with the WHO Ethical Criteria for Medicinal Drug Promotion [22] nor with self-regulatory standards [23]. The overall compliance results seem somewhat more positive than those obtained in the Dutch study [21], but 58.6% of the campaigns included in our Latvian sample failed to comply with two or more of these key criteria.

According to the WHO *‘promotion’ includes all informational and persuasive activities of manufacturers and distributors, that affect the prescription, supply, purchase and/or use of medicinal drugs"* [22]. Although disease awareness campaigns can contain information which might be of potential value to the public, they also have many characteristics that would make them promotional and are, in some cases, clearly designed to support treatment with a sponsor’s product as part of a marketing campaign. They are not subject to the same type of regulatory oversight in Latvia as other types of pharmaceutical promotion. Generally, if the product name is not mentioned, these are not considered to be pharmaceutical advertising, even if the sponsor has a product on the market to treat the condition that is under discussion.

In 78% of the cases we were unable to identify the author or sponsor of the campaigns. This means that the target audience might remain unaware of the intent of the information conveyed [5] and of its potential commercial source. Ebeling describes ‘condition branding’ as an essential component of direct-to-consumer marketing of pharmaceuticals in the United States, with the definition of symptoms associated with a specific treatment being a key focus of activities aiming to create a market for newly developed products [31]. In a randomized trial of a fictitious advertising campaign, consumers tended to perceive disease awareness campaigns more positively than branded advertisements, and stated their intent to seek information and treatment more often after viewing disease awareness campaigns [32].

Some of the conditions mentioned in non-compliant campaigns in our sample have been highlighted in the medical literature as subject to overdiagnosis: female sexual dysfunction, overactive bladder, erectile dysfunction, nail fungus, seasonal affective disorder and excessive sweating [17, 33]. Under the guise of education, companies define conditions and their associated symptoms in the minds of physicians and patients while predicating the best available treatment [31].

The information provided in the disease awareness campaigns collected in our study was often incomplete or misleading about the presentation of benefits and harms of medicines and lacked balance. Prevalence rates were often inaccurate and suggested nearly everyone had the health problem, such as a 90% cited rate of neck and back pain (Table 2). Our results are consistent with reports in other settings [34, 35] of striking statistics, exaggerated stated incidence, prevalence or condition severity [36] [37]. They also mirror existing evidence of the display of striking visuals [1] and use of emotive messages to build brand loyalty [20]. Inaccuracies and information imbalance can lead to increased health care costs if new more expensive drugs are used instead of equally effective lower-cost drugs or non-drug treatments, and even to avoidable injury or death if patients are encouraged to ask for drugs that are less safe than alternatives [38]. For serious conditions, an additional concern is that patients may seek less effective treatments, again leading to avoidable harm.

We found many non-compliant websites in disease awareness campaigns. Websites pose several challenges to regulators, including difficulties ascertaining the source of available information, frequently changing content, and global access to websites that are covered by differing national regulations, including those originating in countries where direct-to-consumer advertising of prescription medicines is legal [5]. Nearly any user worldwide can encounter unregulated and unmonitored pharmaceutical information online [1].

Concerns have been raised about campaigns’ potential to exaggerate the risks of a condition, which may result in increased anxiety and unnecessary visits to doctors [37]. When adoption of newer more expensive products without established advantages over cheaper alternatives is encouraged, this can lead to more doctor visits and inappropriate prescribing [17, 39, 40], shifting both the quality and the costs of care [3]. This is particularly critical in Latvia with its under-resourced health system [24]. Latvian public expenditure on health is remarkably low when compared with neighbouring countries with similar economic development, and out-of-pocket payments are amongst the highest in the European Union [24]. This might explain why 14% (if not more) of the disease awareness campaigns were sponsored by private clinics. The commercial imperative behind these campaigns may be fuelling otherwise unnecessary diagnostic testing and treatment. Such strategies do not comply with the WHO Ethical Criteria which clearly outlines that promotional activities should *“not take undue advantage of people’s concern for their health”* [22].

As the implementation of the WHO Ethical Criteria remains incomplete across the world, researchers have called for an update, claiming that many new marketing strategies are not adequately covered by the 1988 document [41]. One of the points highlighted by researchers is the need to expand the document to include a broader range of ethical values, providing also details on how to interpret and act upon them. Our methodology offers another approach, showing that the existing principles can be interpreted and applied into a practical tool enabling further scrutiny of promotional materials distinguishing legitimate awareness campaigns from covert unbranded advertising.

We found several disease awareness campaigns that referred directly to a specific brand-name drug or indirectly to a treatment but which also included the company’s name or logo, in contravention of the EU directive prohibiting direct-to-consumer advertising of prescription-only medicines. The Latvian Health Inspectorate reported that 15% of the contraventions to advertising regulation in 2016 were direct-to-consumer advertisements for prescription-only medicines [42]. However, despite this experience, the Inspectorate does not actively monitor disease awareness campaigns. There is a contradiction between prescription-only status, requiring provision by clinicians with specialised training and knowledge, and allowing those same drugs to be marketed to people who lack that specialised knowledge [43]. The overall lack of compliance with current international and European standards points to the need for more active monitoring and enforcement. One strategy at hand would be the development and implementation of specific guidance on disease awareness campaigns and communication on prescription-only medicines similarly to what has happened in the United Kingdom and in the Netherlands [23].

Our study had some limitations. As the study period covered 6 months, seasonality is likely to have influenced the content of campaigns. We would not expect an effect on quality, however. As one of our aims was to probe the campaigns’ compliance with the guidelines, we opted to include all types of campaigns. Not all were necessarily sponsored by pharmaceutical companies. One of the striking findings was that the name of the sponsor was not included in 78% of the cases, thus we were unable to clearly identify the subset which was directly or indirectly supported by pharmaceutical companies. Due to this limitation, we were also not able to assess whether the sponsor had ratified or not self-regulatory guidelines. Additionally, while we did not use the instrument to assess news items, we found some features consistent with drug promotion in news coverage of specific conditions and new treatments, such as use of key opinion leaders and lack of information balance. Further application of the instrument in other jurisdictions could shed light on the enforcement status of disease awareness campaigns and inform future policy about adequate measures to respond to the challenges raised by this type of promotional activities.

## Conclusion

Disease awareness campaigns are present in Latvian printed and online media. Their compliance with international and European regulatory standards (namely EU law and pharmaceutical industry self-regulatory guidelines) is low. This raises concerns about the nature of information being conveyed. Through lack of balance, missing sponsorship information, and misleading or incomplete information, these campaigns could contribute to inaccurate self-diagnosis and generate demand among those who might not need medical treatment.

We have developed an instrument to systematically evaluate the information content of disease awareness campaigns. The use of this instrument may help identify promotional campaigns and encourage the effective monitoring and implementation of the regulations.

## Additional files

Additional file 1: **Table S1.** Data collection. List of publications included. (DOCX 16 kb)

Additional file 2: **Table S2.** Overlap between WHO Ethical Criteria, Dutch Self-regulatory guidelines and the instrument. **Table S2.** Provides an overlap between relevant provisions within the WHO Ethical Criteria for Medicinal Drug Promotion and the Dutch Self-Regulatory (CGR) Guidelines for provision of information on prescription medicines and the relevant sections of the instrument. (DOCX 116 kb)

## Abbreviations

DTCA: Direct-to-consumer advertising
EU: European Union
WHO: World Health Organization
