# Supplementary material for: Disease awareness campaigns in printed and online media in Latvia: cross-sectional study on consistency with WHO ethical criteria for medicinal drug promotion and European standards
Source: BMC Public Health. 2018 Nov 28;18:1322. doi: 10.1186/s12889-018-6202-2 (PMC6263056; doi:10.1186/s12889-018-6202-2)
Supplement: Supplementary file 1 — Table S1. Data collection. List of publications included. (DOCX 16 kb) [file 12889_2018_6202_MOESM1_ESM.docx]

**Table S1: Data collection - List of publications included**

| **Format** | **Title** | **Type of Media** | **Language/frequency** |
| --- | --- | --- | --- |
| **Print** | Latvijas Avīze (Latvian Newspaper)  Diena (Daily) | Newspapers | LV daily  LV daily |
|  | MK Latvija (MK Latvia) | Newspaper | RU daily |
|  | Privātā dzīve (Private Life)  Kas Jauns (What’s New) | Tabloids | LV weekly  LV weekly |
|  | Rīgas Santīms (Riga’s Santim) | Free | RU weekly |
|  | Santa (Santa)  Klubs (Club) | Lifestyle magazines | LV monthly  LV monthly |
|  | Lilit (Lilita) | Lifestyle magazine | RU monthly |
|  | Ievas veselība (Ieva’s Health)  Ko ārsti tev nestāsta (What doctors Don’t Tell You) | Health magazines | LV bi-weekly  LV monthly |
|  | Vesti Segodnja Pro Zdorovje (Health News today) | Health magazine | RU monthly |
| **Online** | Delfi.lv | News portal | LV |
|  | Tvnet.lv | News portal | LV |
|  | Vesti.lv | News portal | RU |
|  | Vesels.lv | Health Information portal | LV |
|  | Medicina.lv ( | Health Information portal | LV |
|  | azbuka.lv | Health Information portal | RU |
