# Supplementary material for: Disease awareness campaigns in printed and online media in Latvia: cross-sectional study on consistency with WHO ethical criteria for medicinal drug promotion and European standards
Source: BMC Public Health. 2018 Nov 28;18:1322. doi: 10.1186/s12889-018-6202-2 (PMC6263056; doi:10.1186/s12889-018-6202-2)
Supplement: Supplementary file 2 — Table S2. Overlap between WHO Ethical Criteria, Dutch Self-regulatory guidelines and the instrument. Table S2. Provides an overlap between relevant provisions within the WHO Ethical Criteria for Medicinal Drug Promotion and the Dutch Self-Regulatory (CGR) Guidelines for provision of information on prescription medicines and the relevant sections of the instrument. (DOCX 116 kb) [file 12889_2018_6202_MOESM2_ESM.docx]

| **Table S2: Overlap between relevant provisions within the WHO Ethical Criteria for Medicinal Drug Promotion and the Dutch Self-Regulatory (CGR) Guidelines for provision of information on prescription medicines and the relevant sections of the instrument** | | | | |
| --- | --- | --- | --- | --- |
|  | WHO Ethical Criteria | Dutch CGR Guidelines | SECTIONS IN THE INSTRUMENT | |
| Key Criteria |  |  | Compliant (C) | Non-compliant (NC) |
| 1.Promotional information | Article 6. Definition of promotion: “all informational and persuasive activities by manufacturers and distributors, the effect of which is to induce the prescription, supply, purchase and/or use of medicinal drugs.”  Article 7. “Promotional material should  not be designed so as to disguise its real nature.”  Article 9. “Scientific and educational activities should not be deliberately used for promotional purposes.”  Article 14b. “Advertisements to the public should not generally be permitted for prescription drugs or to promote drugs for certain serious conditions that can be treated only by qualified health practitioners.” | Introduction. Definition of promotion: “all informational and persuasive activities by manufacturers and distributors, the effect of which is to induce the prescription, supply, purchase and/or use of medicinal drugs.”  Introduction. “Instances whereby prescription medication or pills are being mentioned without indicating the drug’s brand name or company name” are considered indirect reference, for example when naming the active ingredients or the drug’s mechanism of action.  Article 5. “Information may not encourage irrational use of prescription medicines nor the search for unnecessary treatment.”  Article 6. “Information may not directly or indirectly lead to the choice of a particular medicine from different available treatments.” | NO (in)direct reference to a pharmaceutical intervention, by for example: Naming a therapeutic class or Naming or displaying a specific medicine or Using a picture and/or link suggesting intervention (ex. “stop-now.com”) or Naming a treatment in general for which only one drug is available | (In)direct reference to a pharmaceutical intervention, by for example: Naming a therapeutic class or  Displaying a specific medicine or Using a picture and/or link suggesting intervention (ex. “stop-now.com”) or Naming a treatment in general for which only one drug is available |
|  |  |  | AND/ OR | IN COMBINATION WITH: |
|  |  |  | NO reference to a pharmaceutical company is made | A reference to a pharmaceutical company OR Naming a drug by brand name (e.g. company’s logo or name mentioned in the text) |
| 2. Misleading or incomplete information | Article 7 . “Advertisements may claim that a drug can cure, prevent, or relieve an ailment only if this can be substantiated. … All promotion-making claims concerning medicinal drugs should be reliable, accurate, truthful, informative, balanced, up-to-date, capable of substantiation and in good taste. They should not contain misleading or unverifiable statements or omissions likely to induce medically unjustifiable drug use or to give rise to undue risks.” | Article 3. “Information may not be misleading. The information provided must comply with the most recent evidence and practice standards. The information must be factually correct and may not contain any misleading elements.”  Article 17. “No comparison is allowed between relevant treatments and medicines that suggests that the effects of a treatment with a prescription drug are better or equal than those of another relevant treatment or drug.”  21.2 b) “No single option for treatment is to be highlighted, for instance by using words, colours or images, different font types, markings or any other elements. ”  Article 21.2 d) “Treaments should be cathegorised based on acceptable formats. For instance using therapeutic classes or categories, or through therapeutic guidelines. Using expressions such as “most recent, or new is better, most commonly used, is not allowed.”  Article 23. “Information should be displayed objectively and neutrally and must not contain information which relates directly to a specific treatment. When reference is made to specific treatment guidelines, the source must be listed…References to scientific literature should also be published…” | The information about pharmaceutical treatment meets national clinical guidelines | The treatment presented is off label and / or does not meet national clinical guidelines |
|  |  |  | OR | OR |
|  |  |  | Both a new therapy AND old therapy (in line with national clinical guidelines) are mentioned. (If the new therapy is the only one mentioned and no suggestion is made about its superiority, select N.A) | A comparison is made between several pharmaceutical treatments, highlighting the superiority of a given treatment, which does NOT meet clinical guidelines. (Additional information: a treatment is portrayed in a positive light and adjectives such as ‘new’, ‘spectacular’ and ‘effective’ are used) |
|  |  |  | Claims or statements made about Prevalence or Incidence or Costs or Disease gravity or Disease burden are accompanied by reference(s) to available evidence (e.g. current guidelines and peer-reviewed journals) | NO reference is provided on the sources of the information provided about: Prevalence or Incidence or Costs or Disease gravity or Disease burden |
| 3. Use of Fear | Article 14: “While they [advertisements] should take account of people’s legitimate desire for information regarding their health, they should not take undue advantage of people’s concern for their health.”  Article 15. “Language which brings about fear or distress should not be used.” | Article 4. “Information should not boost or amplify feelings of fear and superstition and should be displayed realistically.”  Article 5. “Information may not encourage irrational use of prescription medicines nor the search for unnecessary treatment.“  Article 9.“Information should not aim nor encourage the public to seek unnecessary treatment, advice or further examination; nor on the other hand refrain the public from seeking treatment, advice or further examination.”  Article 20. “The information may not be unjustified, unnecessarily alarming or misleading images of changes to the human body resulting from illness or disease. | There is NO reference to fatal events or disability caused by not treating the disease (through a pharmaceutical intervention) | The text and/ or a picture refers fatal events or disability resulting from the non-treatment on the disease |
| 4. Inadequate Language | Article 29. “The wording …if prepared specifically for patients, should be in lay language on condition that the medical and scientific content is properly reflected.” | Article 7. “Information should be tailored to the average consumer and have understandable language. Medical and scientific terms should be avoided as much as possible, to avoid confusion.” | Medical and scientific terminology are correctly described and interpreted. | Use of medical and scientific terminology without providing a (correct) explanation or interpretation |
| 5. Lack of Balance | Article 7. “All promotion-making claims concerning medicinal drugs should be reliable, accurate, truthful, informative, balanced, up-to-date, capable of substantiation and in good taste. They should not contain misleading or unverifiable statements or omissions likely to induce medically unjustifiable drug use or to give rise to undue risks… Comparison of products should be factual, fair and capable of substantiation”. | Article 9.“Information should not aim nor encourage the public to seek unnecessary treatment, advice or further examination; nor on the other hand refrain the public from seeking treatment, advice or further examination.”  Article 17. “No comparison is allowed between relevant treatments and medicines that suggests that the effects of a treatment with a prescription drug are better or equal than those of another relevant treatment or drug.”  Article 21. “Information should be as balanced and complete as possible. It should reflect the state-of-the-art. When providing information, all relevant factors should be taken into account. All information should be equally displayed both in content and layout, with the same amount of detail.”  Article 21.2 c) “The positive and negative effects of a treatment are not to be emphasized in such a way that the pros or the cons of a given treatment are highlighted”.  Article 21.2 d) Information about different therapeutic interventions can be provided. In that case, all relevant treatments should be named, including pharmacotherapy and other interventions, such as adjustments to lifestyle, nutrition and habits. Relevant treatments are the standard of care provided, as per treatment guidelines. Completeness ensures that no information is deliberately omitted. When enumerating all the pharmacotherapeutic options for treatment, all the relevant prescription drugs for the specific treatment are to be mentioned.” | Treatment benefits and harms are accurately and proportionally portrayed. (Additional information: Beneﬁts referred can include symptom control or elimination, prevention of recurrence, or eliminating disease. Harms/Risks can include side effects, complications and adverse drug reactions) | More emphasis on the benefits of pharmaceutical treatment than on its risks. (Additional information: Beneﬁts referred can include symptom control or elimination, prevention of recurrence, or eliminating disease. Harms/Risks can include side effects, complications and adverse drug reactions) |
|  |  |  | Sufficient and correct information is provided to clearly distinguish between a condition requiring drug treatment and normal health and/or milder conditions not requiring drug treatment | Non-pharmaceutical interventions are erroneously omitted |
|  |  |  |  | OR |
|  |  |  |  | Risk factors are portrayed as disease(s) |
|  |  |  |  | OR |
|  |  |  |  | Natural ageing processes such as osteoporosis (at 50 +), menopause, arteriosclerosis etc. are portrayed as disease(s) |
|  |  |  | Symptoms and/or treatment are not emphasized | Symptoms and/ or treatment are accentuated by layout and/ or enumeration |
| 6. Use of Testimonials | Article 7. “Promotional material should not be designed so as to disguise its real nature.”  Article 9. “Scientific and educational activities should not be deliberately used for promotional purposes.” | Article 18. “Testimonials should portray the opinion or experience of the user truthfully (not that of a professional or any other public figure). They should not include any comparison of the user’s situation before and after drug treatment…Before/after testimonials are not allowed because they can lead the public into false expectations regarding the speed of the treatment’s effects”. | There is no professional, scientist or public figure supporting the treatment with a specific drug | The opinion or experience of a professional, scientist or a public figure is given in support of treatment with a specific drug |
|  |  |  | AND/ OR | AND/ OR |
|  |  |  | NO before / after treatment comparison involving an individual patient | A comparison is made of the patient’s experience before and after treatment with a specific drug |
| 7. Absence of Author/sponsor | Article 7. “Advertisements may claim that a drug can cure, prevent, or relieve an ailment only if this can be substantiated. … All promotion-making claims concerning medicinal drugs should be reliable, accurate, truthful, informative, balanced, up-to-date, capable of substantiation and in good taste. They should not contain misleading or unverifiable statements or omissions likely to induce medically unjustifiable drug use or to give rise to undue risks.” | Article 22.“Each message is to contain the name of the person responsible for the information”.  Article 23. “Information may refer to scientific studies and results…The source must always be included. The studies and the results that are mentioned must always come from other sources than the medicine’s producer and should be verifiable…” “Information should be displayed objectively and neutrally and must not contain information which relates directly to a specific treatment. When reference is made to specific treatment guidelines, the source must be listed…References to scientific literature should also be published…” | The author and/or sponsor is /are clearly stated. | The author and/or sponsor is/are not mentioned. |
